# Seroprevalence and risk factors of brucellosis in livestock in the wildlife and livestock interface area of Similipal Biosphere Reserve, India

**DOI:** 10.14202/vetworld.2020.465-470

**Published:** 2020-03-13

**Authors:** Sujit Kumar Behera, Deepanker Das, K. Balasubramani, Savitha Chellappan, Kaushik Rajaram, Himanshu Kumar Mohanta, Praveen Balabaskaran Nina

**Affiliations:** 1 Department of Epidemiology and Public Health, Central University of Tamil Nadu, Tiruvarur, Tamil Nadu, India; 2 Department of Geography, Central University of Tamil Nadu, Tiruvarur, Tamil Nadu, India; 3 National Institute of Traditional Medicine (ICMR), Belgaum, Karnataka, India; 4 Department of Microbiology, Central University of Tamil Nadu, Tiruvarur, Tamil Nadu, India; 5 Block Veterinary Officer, Bangriposi Veterinary Dispensary, Mayurbhanj, Odisha, India

**Keywords:** binary logistic regression analysis, bivariate analysis, brucellosis, rose bengal plate test, seroprevalence

## Abstract

**Background and Aim:**

Brucellosis is an important zoonotic disease that affects fertility in farm animals. The risk factors of brucellosis have not been well studied. This study aimed to understand the seroprevalence and risk factors of brucellosis among livestock in Bangriposi block of Mayurbhanj district in Odisha, a region that borders Similipal wildlife reserve.

**Materials and Methods:**

Rose Bengal plate test (RBPT) was carried out to estimate the seroprevalence of the livestock in this region. Bivariate analysis was carried out to analyze the association between the variables and brucellosis. Binary logistic regression was performed to assess the risk factors associated with brucellosis in the livestock.

**Results:**

Based on RBPT, the seroprevalence of brucellosis among cattle and goats was estimated to be 1.1% and 11.2%, respectively. Binary logistic regression analysis indicates that study area, age, goats, animals with a history of abortion, and rearing practices were the major risk factors in this region.

**Conclusion:**

This is one of the first studies in India to shed light on risk factors of brucellosis, an important neglected disease that affects the health of animals and humans and nation’s economy.

## Introduction

Brucellosis is an important zoonotic disease affecting the health of domesticated animals and humans [1], and as of now, 12 species of *Brucella* have been identified affecting a wide range of vertebrate hosts [2]. *Brucella abortus* and *Brucella melitensis* are the major cause of bovine and caprine brucellosis, respectively [1], and in their natural hosts, the major manifestation of the disease is abortion and infertility [3,4]. Among the species that cause human infection, *B. melitensis* is the most virulent [1]. Worldwide, an estimated 500,000 cases of human brucellosis occur every year [2]. The major routes of human transmission include contact with animal secretions, consumption of raw dairy products, and undercooked meat [3]. In India, an estimated 80% of the population live in close contact with domestic or wild animals and are at risk of acquiring brucellosis [5]. In humans, *Brucella* causes acute and chronic infections, and a lack of awareness may lead to misdiagnosis [3,5]. Several studies have reported human brucellosis in India, and the prevalence is high in individuals who are in close contact with farm animals [3,5].

Indian Government has recently launched in September 2019 a National Animal Disease Control Programme for foot and mouth disease (FMD) and brucellosis. The brucellosis control program aims to provide 100% vaccination coverage to 3.6 crore female calves through the calfhood vaccine S19. In India, screening of livestock for seroprevalence of brucellosis is routinely carried out by Project Directorate on Animal Disease Monitoring and Surveillance (PD_ADMAS), one of the key centers working on zoonotic diseases under the Indian Council of Agricultural Research. A nationwide survey of cattle and buffalo carried out from 1994 to 2001 by PD_ADMAS found seropositivity of 5% in cattle and 3% in buffalo [6,7]. A recent 2019 report estimates seropositivity of 8.3% and 3.6% in cattle and buffaloes, respectively [8], suggesting >60% increase in *Brucella* prevalence in cattle. Caprine brucellosis is endemic in India [7], and in many countries in the Middle East, the Mediterranean region, Central Asia, countries in sub-Saharan Africa, and some parts of Latin America, where small ruminants are the major source of livelihood [9]. In India, depending on the geographical location, farms, animal husbandry practices, and health status, the seroprevalence of caprine brucellosis varies greatly [10-14]. A nationwide survey carried out in 1994-1998 by PD_ADMAS found seropositivity of 7.9% in sheep and 2.2% in goat.

Epidemiological studies carried out in different geographical locations have identified age, herd size, presence of small ruminants, herd composition, animal husbandry practices, and socioeconomic factors to be the major risk factors of brucellosis [15,16].

Despite the widespread prevalence of brucellosis among farm animals in India, the major risk factors that precipitate brucellosis are poorly understood. To understand *Brucella* seroprevalence and risk factors, we carried out a study in the foothills of Similipal Biosphere Reserve, Mayurbhanj district of Odisha in the interface between forest and domestic habitation.

## Materials and Methods

### Ethical approval

Ethical approval is not applicable for this study.

### Study area, period and design

The study area is Bangriposi block in Mayurbhanj district near the periphery of Similipal Biosphere Reserve. The samples were obtained from two major clusters. One is the Kusumbandha and neighboring sites of Astabeda, Needam, Palasbani, Kashadihi, Rangamatia, and Kamalpur. The other cluster is the Bhusani and the neighboring sites of Kirpaduma, Hindusahi, and Matiali Sahi. A cross-sectional study was carried out from January to March 2019 to understand the epidemiology of brucellosis in the Bangriposi block. The serum samples from livestock that were used in this study were collected as part of a regular FMD surveillance program carried out by the veterinary dispensary of the Bangriposi block under the Assistance to States for Control of Animal Disease program of Government of Odisha. The livestock owners were asked questions about their livestock and livestock rearing practices**.** The locations of livestock owners were captured using handheld Global Navigation Satellite System for understanding the spatial relationships.

### Serum collection

The blood samples (3-4 ml) were collected by jugular venipuncture from animals through a disposable syringe and were stored in a Vacutainer without any anticoagulant. The blood was allowed to clot overnight. Next day morning, the separated serum was collected in a microcentrifuge tube. The microcentrifuge tube was coded and was stored at −20°C. The blood samples were collected from 485 animals.

### Rose Bengal plate test (RBPT)

RBPT was carried out using standard RBPT antigen (*B. abortus* S99 strain) obtained from the Institute of Animal Health and Veterinary Biologicals, Bengaluru, India, according to the method of Alton *et al*. [17]. Equal volumes (30 µl) of antigen and test serum were mixed thoroughly on the glass plate using a toothpick, and the mixture was gently agitated or rocked for 3-4 min at room temperature. Any agglutination (observed as spots, flakes, or dotted particles) was considered as a positive reaction.

### Statistical analysis

The variables related to the livestock such as age, sex, place, type of livestock, herd composition, herd size, history of aborted fetus, disposal of aborted fetus, retained placenta, disposal of retained placenta, method of rearing methods (modern/traditional), grazing source, and contact with other herds were described using counts and percentages. Bivariate analysis was carried out to analyze the association between the variables and brucellosis. We used the Pearson’s Chi-square test or Fisher’s exact test as required.

Binary logistic regression was performed to assess the risk factors associated with brucellosis in the livestock. Seropositivity to brucellosis (yes/no) was the binary dependent variable. Independent variables used were the type of livestock, sex, age, place, history of aborted fetus, and rearing methods. Most of the independent variables included in the regression model were significant in the bivariate analysis. Adjusted odds ratio (aOR) was calculated along with the confidence intervals (CI).

Statistical significance was based on p<0.05. Data were analyzed using SPSS version 22 (IBM Corp., NY, USA). MS Excel 2013 was used to enter data, make tables and graphs. ArcGIS 10.5 was used to prepare the base map of the study area.

## Results

### Seroprevalence of brucellosis

**Figure-1:**
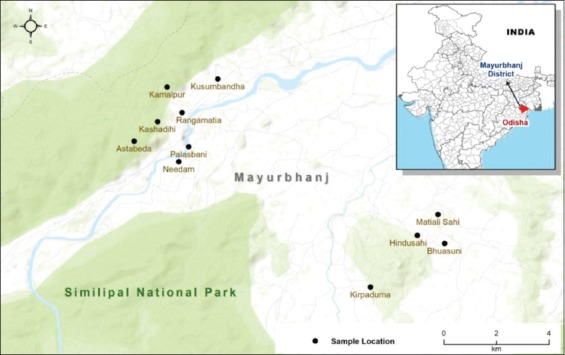
Base map of the study area. The dark dots represent the sample locations. The light green color indicates the extent of reserve forest (Similipal National Park). The inset map shows the location of Mayurbhanj district, Odisha [Source: Map was prepared by the authors].

**Figure-2:**
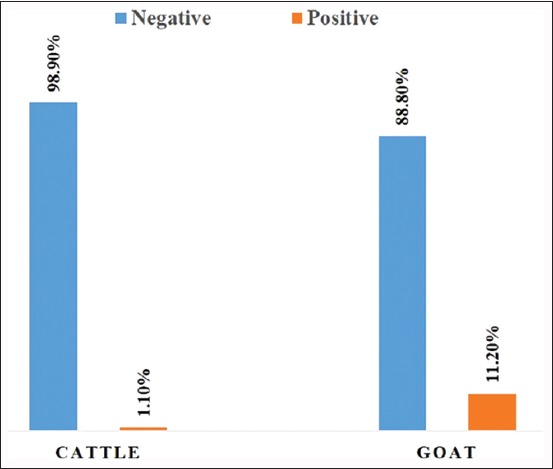
Seroprevalence of brucellosis in goats and cattle. Blue bar indicates negative (%). Orange bar indicates positive (%).

The study was carried out in the Bangriposi block, near the periphery of Similipal National Park in Mayurbhanj district of Odisha (Figure-1). The seroprevalence of brucellosis among cattle and goats (n=475) in the studied sites of Mayurbhanj district at Odisha was 9.3% (95% CI: 6.65-11.87). The seroprevalence of brucellosis among goats and cattle was 11.2% (43 positives out of 385) and 1.1% (1 positive out of 90), respectively (Figure-2). The variables associated with the seroprevalence of brucellosis are given in Table-1. The seroprevalence of brucellosis was significantly higher in animals greater than 10 years of age (7.7% in 1-5 years age group, 8.9% in 6-10 years age group, and 26.7% in >10 years). Age differences in the seroprevalence of brucellosis were statistically significant. The seroprevalence of brucellosis was significantly higher in Kusumbandha area (12.5%) than in Bhuasuni area (0.8%). The seroprevalence in goats (11.2%) was significantly higher compared to cattle (1.1%). Livestock with a history of aborted fetus had significantly higher seroprevalence (22.2%) compared to livestock without a history of aborted fetus (8.5%). Livestock reared using modern methods had higher seroprevalence (26.1%) compared to traditional methods (8.4%), and the difference is statistically significant.

**Table-1:** Seroprevalence of brucellosis among goats and cattle by sociodemographic and husbandry practices.

Variables	Brucellosis		χ^2^ (p-value)

	Negative (%)	Positive (%)	
Study area			
Bhuasuni	131 (99.2)	1 (0.8)	15.734 (0.000)
Kusumbandha	300 (87.5)	43 (12.5)	
Livestock species			
Cattle	89 (98.9)	1 (1.1)	8.779 (0.003)
Goat	342 (88.8)	43 (11.2)	
Sex			
Male	224 (91.1)	22 (8.9)	0.062 (0.803)
Female	207 (90.7)	22 (9.6)	
Abortion			
Yes	21 (77.8)	6 (22.2)	5.720 (0.017)
No	410 (90.7)	38 (8.5)	
Retained placenta			
Yes	17 (85.0)	3 (15.0)	0.818 (0.366)
No	414 (91.0)	41 (9.0)	
Management			
Modern	17 (73.9)	6 (26.1)	8.139 (0.004)
Traditional	414 (91.6)	38 (8.4)	
Disposal of aborted fetus			
NA	309 (90.6)	32 (9.4)	4.116 (0.128)
Buried	94 (94.0)	6 (6.0)	
Thrown	28 (82.4)	6 (17.6)
Grazing source			
Common grazing field	22 (100)	0 (0)	2.355 (0.125)
Jungle land	409 (90.3)	44 (9.7)	
Contact with other herds			
Yes	384 (90.8)	39 (9.2)	0.009 (0.926)
No	47 (90.4)	5 (9.6)	
Herd size			
1-15	323 (91.8)	29 (8.2)	2.542 (0.281)
16-30	60 (85.7)	10 (14.3)	
30+	48 (90.6)	5 (9.4)	
Age (years)			
1-5	286 (92.3)	24 (7.7)	11.687 (0.003)
6-10	123 (91.1)	12 (8.9)	
10+	22 (73.3)	8 (26.7)	
Disposal of retained placenta			
Buried	100 (95.3)	7 (6.5)	5.769 (0.056)
NA	303 (91.0)	30 (9.0)	
Thrown	28 (80.0)	7 (20.0)	

NA=Not available

### Risk factors of brucellosis

Logistic regression analysis showed that increasing age, Kusumbandha area, goats, history of aborted fetus, and modern rearing methods are major risk factors associated with brucellosis (Table-2). Increasing age is a major risk factor associated with brucellosis. Animals greater than 10 years had the highest risk (aOR=4.1, CI: 1.5-11.1) of brucellosis followed by livestock in the age group of 6-10 years when compared to livestock in the age group of 1-5 years. Livestock in Kusumbandha and neighboring sites is 11.9 times more likely to be *Brucella* seropositive compared to Bhuasuni and its surrounding sites (CI: 1.6-90.1). Goats are 11.8 times more likely to be *Brucella* seropositive than cattle (CI: 1.4-98). Livestock with a history of aborted fetus is 3 times at higher risk of acquiring brucellosis when compared to livestock without a history of aborted fetus (CI: 1-8.7). Rearing the livestock using traditional methods was shown to be associated with a lower risk of brucellosis (aOR=0.27, CI: 0.09-0.82), compared to modern rearing methods.

**Table-2:** Factors associated with brucellosis among goats and cattle.

Factor	β coefficient	SE	OR	95% CI for OR		p-value

				Lower	Upper	
Study area						
Bhuasuni	0	0	1	-	-	
Kusumbandh	2.48	1.03	11.99	1.59	90.16	0.016
Livestock species						
Cattle	0	0	1	-	-	
Goat	2.47	1.08	11.81	1.423	98.05	0.022
Sex						
Female	0	0	1	-	-	
Male	0.25	0.35	1.28	0.65	2.51	0.472
History of abortion						
No	0	0	1	-	-	
Yes	1.11	0.54	3.04	1.06	8.71	0.039
Rearing method						
Modern	0	0	1	-	-	
Traditional	−1.3	0.56	0.27	0.09	0.82	0.021
Age						
1-5	0	0	1	-	-	
6-10	0.35	0.40	1.42	0.64	3.11	0.387
10+	1.41	0.51	4.10	1.52	11.11	0.005
Constant	−6.02	1.51	-	-	-	-

SE=Standard error, OR=Odds ratio, CI=Confidence interval

## Discussion

Brucellosis is a neglected zoonotic disease in developing countries affecting livestock and humans [18]. In addition to affecting health, brucellosis also causes substantial economic loss. One report estimates an annual median loss of USD $ 3.4 billion (5^th^-95^th^ percentile 2.8-4.2 billion) due to brucellosis in animals [8].

Brucellosis could be diagnosed by many serological tests; the widely used are indirect enzyme-linked immunosorbent assay (I-ELISA), complement fixation test (CFT), and RBPT. A Bayesian analysis showed that I-ELISA has the best accuracy followed by CFT and RBPT. However, the decision on the choice of diagnostic test not only should rely on the accuracy but also should consider the time, technical difficulty, and cost-effectiveness. Despite its lower sensitivity, RBPT remains the widely used screening test due to its ability to produce quick results and cost-effectiveness [19]. A serological comparison of RBPT and standard tube agglutination test in screening small ruminant brucellosis showed that their specificity is the same [20].

RBPT was used to investigate the seroprevalence and risk factors of brucellosis in livestock at Mayurbhanj district in Odisha, India. This region lies in close proximity to the Similipal National Park, a wildlife reserve, and this is the first *Brucella* seroprevalence carried out in this area. The seroprevalence in cattle and goats was 1.1% and 11.2%, respectively. The prevalence of brucellosis among livestock varies widely across India’s diverse agricultural landscape, states, and farms [5]. In a mass survey carried out by PD_ADMAS across many states in India during 1994-2001, the seroprevalence of brucellosis was 5% and 3% in cattle and buffalo, respectively [6]. However, the seroprevalence of brucellosis in cattle in the state of Odisha was 1% [6,7] and is comparable to the current study where the seroprevalence was 1.1%. A recent report published by PD_ADMAS in 2019 on *Brucella* survey carried out in 15 states in India has reported a seroprevalence of 8.3% and 3.6% in cattle and buffaloes, respectively, and for cattle in Odisha, it was 1.95% [21]. Overall, in Odisha, *Brucella* seropositivity among cattle is less when compared to other states in India [6,7,21].

A national survey carried out in small ruminants from 10 states has reported a prevalence of 2.2% and 7.9% in goats and sheep, respectively [7]. The brucellosis seroprevalence of goats in the current study was 11.2%, much higher when compared to the national average. However, there is a wide variation in brucellosis seropositivity in goats based on the geographical location in India [10-14,22,23]. In Sudan and Egypt, seroprevalence in goats reported using serological techniques was 11.2%, similar to our findings [24,25].

In the current study, one of the risk factors for brucellosis is the Kusumbandha study area, and animals from this area had a higher risk of acquiring brucellosis than from Bhuasuni area. A probable reason could be the higher number of samples (72.2%) collected from Kusumbandha area. Furthermore, this area is in close proximity to Similipal wildlife reserve, and there is a chance for greater contact with wild animals. The age of animals is also an important risk factor associated with brucellosis seropositivity and is in agreement with the previous studies [26,27]. Sex was not a risk factor of brucellosis in this study, however, an earlier study from Pakistan has showed that females have higher odds of becoming seropositive than males [28]. Studies have shown that herds with a history of abortions have a higher risk of brucellosis [29,30], as reported in this study. Furthermore, compared to cattle, goats were at higher risk of brucellosis and are in line with an earlier study from Jordan [31]. Overall, study area, age, animals with a history of abortion, goats, and animal husbandry practices were the major risk factors for brucellosis in this study. Furthermore, the modern method of rearing (semi-intensive or intensive) where animals are mostly kept indoors and are in close contact with each other is at a higher risk of brucellosis.

The high prevalence of caprine brucellosis is a serious concern due to its zoonotic potential. Most of the livestock owners in this region are illiterate and are not aware of the zoonotic threat of brucellosis. Future studies should address the seroprevalence of human brucellosis in this community. Furthermore, outreach programs should be organized to improve *Brucella* awareness in the community.

## Conclusion

We report the seroprevalence of brucellosis in cattle and goats in the wildlife and livestock interface area of Similipal Biosphere Reserve, Odisha. The high seroprevalence of caprine brucellosis is an important One Health concern. This is one of the first studies in India to shed light on risk factors of brucellosis, an important neglected disease that affects the health of animals and humans and nation’s economy.

## Authors’ Contributions

SKB, HKM, and PBN designed the study. SKB and PBN drafted the manuscript. SKB carried out the RBPT. KB did spatial mapping. DD, SC and KR helped in biostatistics analysis. All authors read and approved the final manuscript.
